# Black Soldier Fly (*Hermetia illucens*) Larvae Meal Modulates Intestinal Morphology and Microbiota in Xuefeng Black-Bone Chickens

**DOI:** 10.3389/fmicb.2021.706424

**Published:** 2021-09-16

**Authors:** Changqing He, Jiaxing Lei, Yaling Yao, Xiangyong Qu, Jifa Chen, Kailai Xie, Xingju Wang, Qi Yi, Bing Xiao, Songchang Guo, Xiaoyan Zou

**Affiliations:** ^1^Hunan Engineering Research Center of Poultry Production Safety, Hunan Agricultural University, Changsha, China; ^2^College of Animal Science and Technology, Hunan Agricultural University, Changsha, China; ^3^Huaihua Animal Husbandry and Fishery Affairs Center, Huaihua, China; ^4^College of Life Science and Resources and Environment, Yichun University, Yichun, China; ^5^Hunan Yunfeifeng Agricultural Co., Ltd., Huaihua, China; ^6^College of Veterinary Medicine, Hunan Agricultural University, Changsha, China

**Keywords:** *Hermetia illucens* larvae meal, Xuefeng black-boned chicken, intestinal morphology, intestinal microbial diversity, gut health

## Abstract

The addition of *Hermetia illucens* larvae meal (HILM) to the feed could contribute to particular antimicrobial and intestinal health in animal husbandry. This study was conducted to investigate the effects of HILM on intestinal morphology and microbial diversity in different intestinal segments of Xuefeng black-bone chickens. All of 432 birds (45 weeks old) were randomly assigned to four equal groups with six replicates and 18 hens in each replicate: (A) basal diet, (B) basal diet with 1% HILM, (C) basal diet with 3% HILM, and (D) basal diet with 5% HILM. The results showed that, compared with the basal diet group, the HILM supplement significantly increased the abundance-based coverage estimator (ACE) and Chao index in cecum (*p* < 0.05). Diet with 1% HILM significantly increased the villus height (VH) of the duodenum (*p* < 0.05) and cecum microbial diversity as represented by the Simpson index (*p* < 0.05). In particular, 1% HILM displayed a markedly increase in the genus unclassified Bacteroidales (cecum, *p* < 0.05). A basal diet with 3% HILM markedly increased the beneficial genus *Romboutsia* (jejunum, *p* < 0.05). Also, principal component analysis (PCA) cluster analysis showed that 3% of HILM was more individual than other groups (*p* < 0.05). However, 5% HILM decreased the VH and the ratio of villus height to crypt depth (VH/CD) of the jejunum and increased beneficial bacteria such as *Staphylococcus* (*p* < 0.05), which was regarded as pathogenetic genera. In conclusion, we found that HILM improved intestinal morphology and increased microbiological diversity and species abundance. Together, dietary supplementation of 1 or 3% HILM might benefit the intestinal morphology and intestinal microbiota of Xuefeng black-bone chicken. However, the addition of 5% HILM could decrease VH and the ratio of VH/CD of the jejunum and increased pathogenetic genera. HILM was an excellent protein substitute for Xuefeng black-bone chickens, which could meet the nutritional requirements under the condition of less feed. These results provide information for HILM meal as an alternative source of soybean meal in Xuefeng black-bone chickens’ feed.

## Introduction

In recent years, the demand for high-quality protein in the world has grown tremendously. Being one of the sources with high-quality protein, it has been reported that human demand for poultry meat would increase more rapidly than any other meat product between 2030 and 2050 (the 25th World’s Poultry Congress, 2016). With this upcoming expansion, the resources needed to grow food and feed were becoming increasingly strained because the production of animal and plant proteins required the use of large amounts of land, water, and energy (Kim et al., 2019; Rathnayaka et al., 2021). Therefore, existing feed resources might not be able to support the current growth in poultry production; alternative feeds and feed protein supplements were absolutely needed to replace the current supply and meet the growing demand (De Smet et al., 2018).

Insect, compared with conventional proteins, seemed to be one of the most promising alternatives (Henry et al., 2015; DiGiacomo et al., 2019; Gravel and Doyen, 2020). *Hermetia illucens* larvae meal (HILM) was characterized by being rich in protein (dry matter >37%) and fat (up to 49%), as well as several macronutrients and micronutrients, which were important for animal development (Smetana et al., 2019). The amino acids of HILM are balanced; most of the amino acid content is similar to fish meals and soybean meals (Barroso et al., 2014). Different from other insects, it was indicated in the prevailing view that HILM products could have particular antimicrobial attributes (Elhag et al., 2017; Fawole et al., 2020; Wang et al., 2020), which might profit from the antimicrobial peptides and chitin in HILM. Furthermore, HILM had a lower environmental impact than other protein sources, making it a potential alternative to regular feed resources for animal production (Wang and Shelomi, 2017). Recently, HILM had been used in the production of livestock (poultry, rabbits, and pigs) and aquaculture species (Gasco et al., 2019).

Xuefeng black-bone chickens, originating from Xuefengshan Mountain and the adjacent region, were the most famous local breed in the Hunan province of China and had been listed as one of the national livestock and poultry genetic resources in China. Being classified into the type of meat-and-egg chicken, Xuefeng black-bone chicken was commonly believed to have medicinal properties and has been used as remedies to enhance the human immune system by providing protein, vitamins, and amino acids (Xie et al., 2020). At present, induced by the switch from quantity to quality of consumers, the market demand for Xuefeng black-bone chicken is increasing (Guo et al., 2021). Moreover, the shortage of protein resources for livestock and poultry feeding drove up the rise of the cost of animal husbandry, which required seeking potential substitutes. Studies have revealed that HILM could be a suitable protein substitution for poultry (Marono et al., 2017; Cutrignelli et al., 2018; Moniello et al., 2019). Nonetheless, its application on Xuefeng black-bone chicken remained poorly understood. In this context, we conducted to explore the effects of HILM on intestinal morphology and microbial diversity in different intestinal segments of the Xuefeng black-bone chicken and provide more detailed information about the capability of HILM as an ideal protein replacement in feed and inform the future diagnosis and therapy of insect meal as a sustainable protein source.

## Materials and Methods

All the animals were humanely conducted in accordance with the principles stated by the Chinese guidelines for animal welfare. The experimental procedures received approval from the animal welfare standards of the College of Animal Science and Technology, Hunan Agricultural University.

### Animals, Diets, and Experimental Design

A total of 432 female Xuefeng black-bone chickens were randomly divided into four groups (6 replicas of 18 hens) and fed for 56 days. The diets based on corn and soybean meal of the chickens were supplemented with 0, 1, 3, or 5% HILM to partly replace soybean meal and designated as A, B, C, and D groups, respectively. The feeding experiment was the same as our previous study (Liu et al., 2021). The experimental diets were isonitrogenous and isoenergetic, but the proportion of HILM was different.

### Sample Collection

At the end of the experiment, three chickens from each group were randomly chosen and killed by cervical dislocation. After killing, the digesta samples from the jejunum and cecum were collected in sterilized plastic tubes and then immediately stored in liquid nitrogen to investigate intestinal microflora. Additionally, segments (10 cm in length) of the middle of the duodenum and jejunum were excised and washed with phosphate-buffered saline until no digesta were visible. The segments were fixed with 4% paraformaldehyde for more than 24 h to make intestinal sections.

### Experimental Parameters Analysis

#### Intestinal Morphology

Five-μm sections were cut from the paraffin-embedded intestinal segments after removing the fixation solution and then stained with hematoxylin and eosin for light microscopy. Zeiss microscope (Axio Imager, A1) and the MShot Image Analysis System were used to take photographs for sections. The villus height (VH) and the crypt depth (CD) were used in five typical fields to measure (Wang and Hogan, 2019).

#### Intestinal Microflora

Genomic DNA was extracted from chyme samples using a kit (DP328, Tiangen Biochemical Technology Co., Ltd., China) (Xia et al., 2020). The samples of qualified DNA were sent to Shanghai Majorbio Bio-pharm Technology Co., Ltd. (China) for polymerase chain reaction amplification and sequencing analysis.

Using quantitative DNA as a template, the 16S rRNA gene V3–V4 region was amplified by primer pairs 338F (5′-ACTCCTACGGGAGCAGAC-3′) and 806R (5′-GGACTACHVGGGTWTCTA-3′). The purified, amplified products were sequenced on the Novaseq PE250 platform (Illumina, San Diego, United States) using equimolar and paired-end sequencing according to MajorBio Biopharma Technologies, Inc. (Shanghai, China) standard protocol.

The original sequence was demultiplexed and quality filtered using FASTP version 0.20.0 (Chen et al., 2018) and merged using Flash version 1.2.7 (Magoc and Salzberg, 2011). The criteria are as follows: (a) the 300-bp reads were truncated at any site receiving an average quality score of <20 over a 50-bp sliding window, and the truncated reads shorter than 50 bp were discarded; reads containing ambiguous characters were also discarded; (b) Only overlap sequences greater than 10 bp are spliced according to overlapping sequences. The maximum mismatch in the overlapping area is 0.2. Reads that cannot be assembled are discarded; (c) samples were distinguished according to the barcode and primers, and the sequence direction was adjusted. The barcode matching was accurate, and the two nucleotides in the primer matching did not match. UPARSE version 7.1 was used to cluster operational taxonomic units (OTU) with a similarity cutoff rate of 97% (Edgar, 2013). RDP Classifier version 2.2 was used to classify each OTU representative sequence, and Silva V132 was used for classification and identification.

### Statistical Analysis

One-way analysis of variance in SPSS 22.0 software (IBM SPSS, United States) was used for the statistical analyses. The analysis results were expressed as the arithmetic mean and standard error. Differences were considered to be significant at *p* < 0.05 and highly significant at *p* < 0.01. The Majorbio Cloud Platform^1^ was used to analyze intestinal microflora.

## Results

### Intestinal Morphology

**FIGURE 1:**
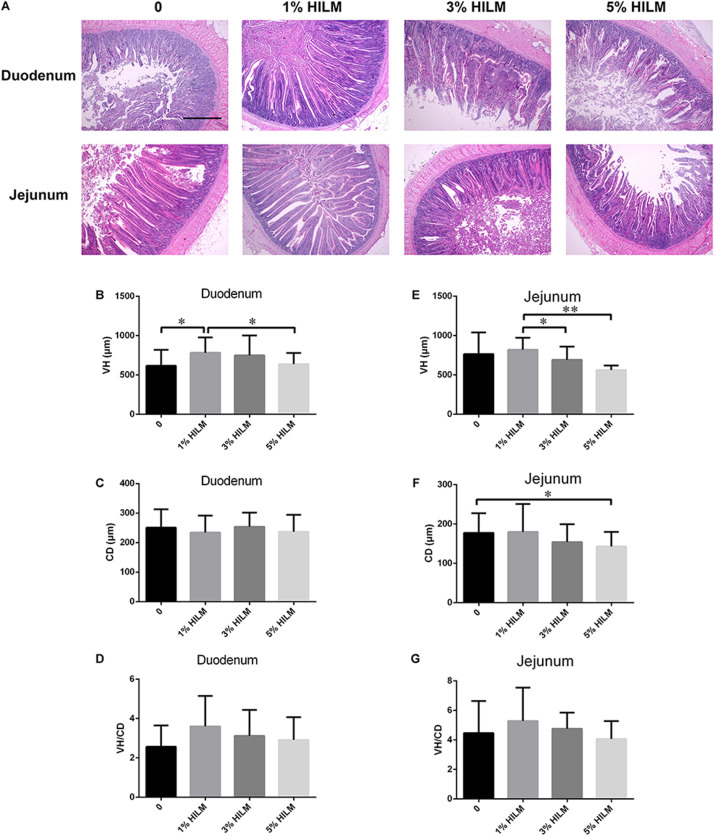
Effects of HILM on intestinal morphology of Xuefeng black-bone chicken. **(A)** Representative pictures of hematoxylin and eosin staining (40×, scale bar: 20 μm); VH of duodenum **(B)** and jejunum **(E)**; CD of duodenum **(C)** and jejunum **(F)**; VH/CD of duodenum **(D)** and jejunum **(G)**. Results shown are means ± SEM (*n* = 12). ^∗^*p* < 0.05, ^∗∗^*p* < 0.01. VH, villus height; CD, crypt depth; VH/CD, ratio of villus height to crypt depth; HILM, *Hermetia illucens* larvae meal.

To determine whether HILM diets were associated with the intestinal morphology of Xuefeng black-bone chickens, we measured the VH and CD of the duodenum and jejunum (Figure 1). In the duodenum, the VH of group B was significantly higher than groups A and D (*p* < 0.05). However, no significant difference was observed in the CD and the ratio of VH to CD (VH/CD) among four treatments (*p* > 0.05). In the jejunum, the VH of group B was the highest, which was markedly higher than groups C and D (*p* < 0.05). The CD decreased significantly in group D, compared with group A (*p* < 0.05).

### Production Performance

The production performance was shown in Table 2 of our previous study (Liu et al., 2021). In the overall period (from day 1 to 56 of the experiment), 3% HILM increased the egg weight (*p* < 0.05), feed intake (*p* < 0.05), and egg production (*p* < 0.05), meanwhile decreased the feed conversion ratio (*p* < 0.05) in a linear or quadratic manner.

### Operational Taxonomic Unit Analysis and Venn Diagram of the Difference in Operational Taxonomic Unit Distributions Between Groups

**FIGURE 2:**
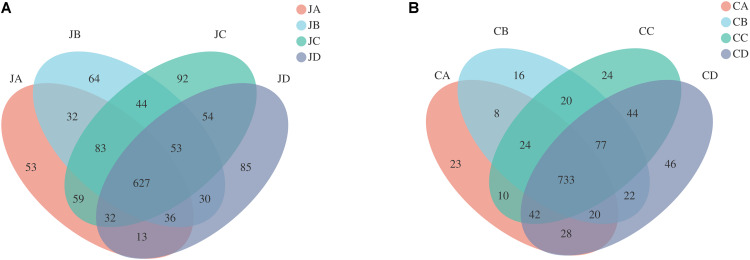
Shared and specific operational taxonomic unit (OTUs) in different intestinal sections of different groups (**A**: jejunum; **B**: cecum). JA, jejunum of 0% HILM; JB, jejunum of 1% HILM; JC, jejunum of 3% HILM; JD, jejunum of 5% HILM; CA, cecum of 0% HILM; CB, cecum of 1% HILM; CC, cecum of 3% HILM; CD, cecum of 5% HILM.

In our study, a total of four groups (each group with two intestinal segments) were investigated, and each sample contained large numbers of enriched OTUs (Figure 2). According to OTU classification, we calculated the same number of OTUs between groups and then pointed them in the Venn graph. Each color block in the Venn graph represents a group, intersecting sections represent OTUs shared with adjacent groups, and one number means one group. The OTUs of the jejunum were as follows (Figure 2A): JA, 53; JB, 64; JC, 92; and JD, 85. Uniform OTUs in the jejunum among groups were as follows: JA and JB, 32; JA and JC, 59; JA and JD, 13; JB and JC, 44; JB and JD, 30; JC and JD, 54; JA, JB, and JC, 83; JA, JB, and JD, 36; JA, JC, and JD, 32; JB, JC, and JD, 53; and JA, JB, JC, and JD, 627. The OTUs in the duodenum were as follows (Figure 2B): CA, 23; CB, 16; CC, 24; and CD, 46. Uniform OTUs in the duodenum among groups were as follows: CA and CB, 8; CA and CC, 10; CA and CD, 28; CB and CC, 20; CB and CD, 22; CC and CD, 44; CA, CB, and CC, 24; CA, CB, and CD, 20; CA, CC, and CD, 42; CB, CC, and CD, 77; and CA, CB, CC, and CD, 733.

### Alpha Diversity

**FIGURE 3:**
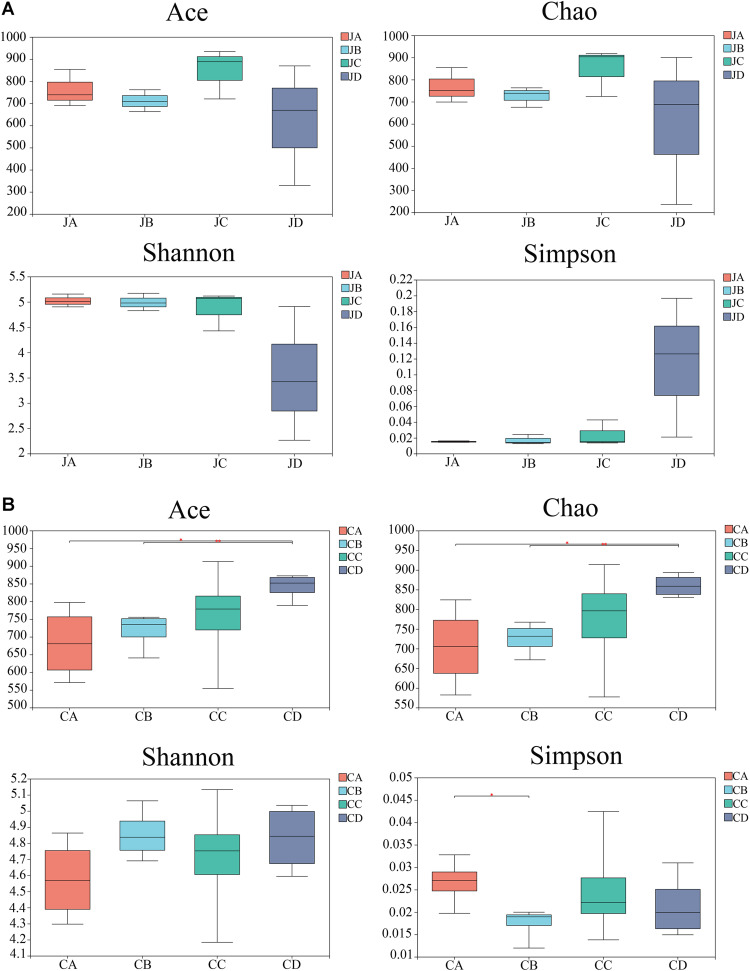
Alpha diversity of four groups in jejunum **(A)** and cecum **(B)**. Significant difference between samples and marks with significant differences between two groups. JA, jejunum of 0% HILM; JB, jejunum of 1% HILM; JC, jejunum of 3% HILM, JD: jejunum of 5% HILM; CA, cecum of 0% HILM; CB, cecum of 1% HILM; CC, cecum of 3% HILM; CD, cecum of 5% HILM.

The abundance-based coverage estimator (ACE) index, Chao index, Shannon index, and Simpson index were selected to analyze diversity (Figure 3). Although dietary HILM did not influence bacterial diversity in the jejunum, the richness of the intestinal microbiota increased in group C, but the community richness and diversity decreased in group D (Figure 3A, *p* > 0.05). However, in the cecum, the ACE index and Chao index of group D were significantly higher than those of group A (*p* < 0.05) and group B (*p* < 0.01). The Simpson index was the lowest, which was significantly higher than that of group A (*p* < 0.05). There were no significant differences observed among the groups in the Shannon index (*p* > 0.05).

### Heatmap Analysis–Phylum Level

**FIGURE 4:**
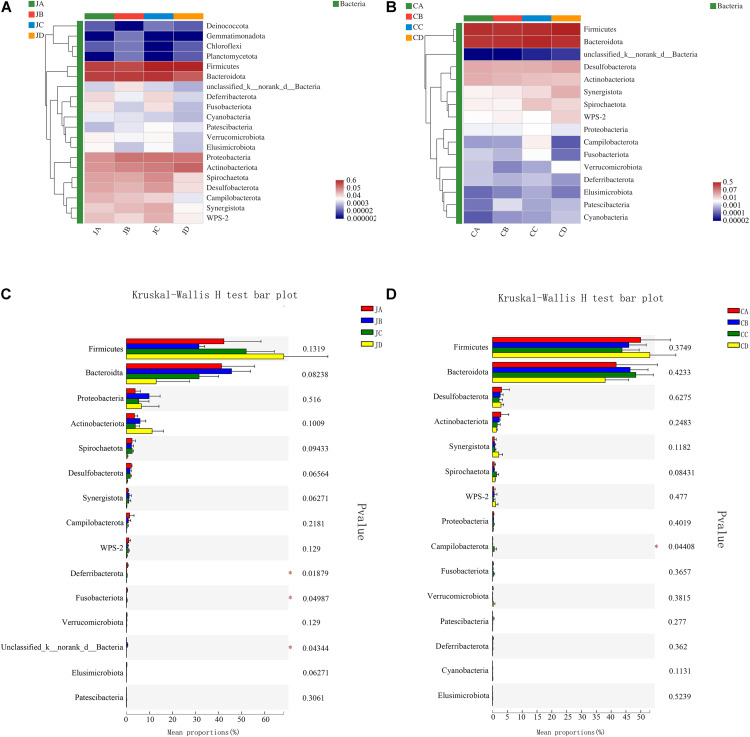
Heatmap chart of top 20 phyla in different intestinal segments (**A**: jejunum; **B**: cecum) and comparison of differences between groups of top 20 phyla (**C**: jejunum; **D**: cecum). One-way analysis of variance, JA, jejunum of 0% HILM; JB, jejunum of 1% HILM; JC, jejunum of 3% HILM; JD, jejunum of 5% HILM; CA, cecum of 0% HILM; CB, cecum of 1% HILM; CC, cecum of 3% HILM; CD, cecum of 5% HILM.

To analyze the effects of HILM on the microbial community, a heatmap was used to assess the composition of microorganisms by color shades among the four groups (each group with two intestinal segments). The results showed that *Firmicutes, Bacteroidetes, Proteobacteria, Actinobacteria, Spirochaetota, Desulfobacterota, Campilobacterota, WPS-2*, and *Synergistetes* accounted for 98.58% of the microbial population (Figure 4A). Among those, *Bacteroidetes* and *Firmicutes* were the main phyla in the jejunum, accounting for 83.37%. As shown in Figure 4C, the *Deferribacterota* and *Fusobacteriota* phyla of group A were markedly improved (*p* < 0.05).

As shown in Figure 4B, the dominant bacterial phyla of the cecum were *Firmicutes, Bacteroidetes, Desulfobacterota, Synergistetes, Spirochaetota*, and *WPS-2*, accounting for 99.12% of the microbial population. Among those, *Bacteroidetes* and Firmicutes were the main phyla in the jejunum, accounting for 91.78%. The *Campilobacterota* phylum of group C was significantly elevated (*p* < 0.05); however, there were no significant differences among other microorganisms (*p* > 0.05, Figure 4D).

### Heatmap Analysis–Genus Level

**FIGURE 5:**
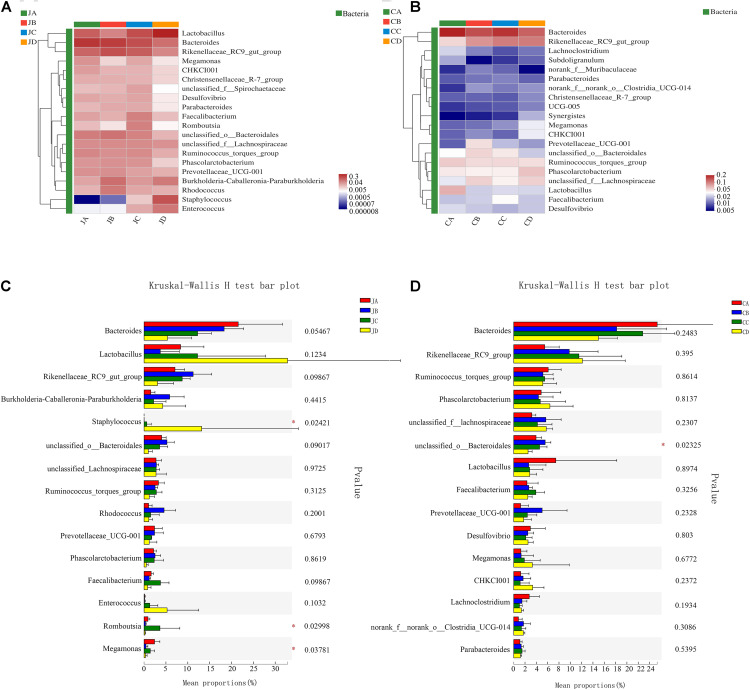
Heatmap chart of the top 20 genera in different intestinal segments (**A**: jejunum; **B**: cecum) and comparison of differences between groups of the top 20 phyla (**C**: jejunum; **D**: cecum). One-way analysis of variance, JA, jejunum of 0% HILM; JB, jejunum of 1% HILM; JC, jejunum of 3% HILM; JD, jejunum of 5% HILM; CA, cecum of 0% HILM; CB, cecum of 1% HILM; CC, cecum of 3% HILM; CD, cecum of 5% HILM.

As shown in Figure 5A, the dominant genus of the jejunum was *Bacteroides*, and the second genus was *Lactobacillus*. At the genus level, the analysis results showed that compared with groups A, B, and C, the abundances of *Staphylococcus* in group D significantly increased. The abundances of *Romboutsia* increased in group C (*p* < 0.05, Figure 5C). However, in group A, we found that the abundances of *Megamonas* were significantly higher than others (*p* < 0.05).

As shown in Figure 5B, the dominant genus of the cecum was *Bacteroides*, and the next genus was *Rikenellaceae*. In group B, we found that the abundances of the unclassified *Bacteroidales* were significantly higher than the other groups (*p* < 0.05, Figure 5D). However, there was no significant change in the intestinal microflora in the cecum (*p* > 0.05).

### Principal Component Analysis Cluster Analysis

**FIGURE 6:**
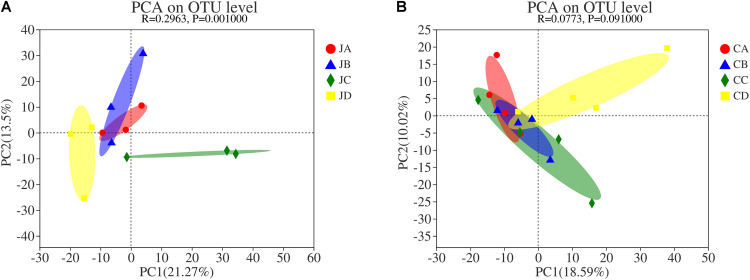
Cluster analysis by principal coordinate analysis. **(A)** jejunum; **(B)** cecum. JA, jejunum of 0% HILM; JB, jejunum of 1% HILM; JC, jejunum of 3% HILM; JD, jejunum of 5% HILM; CA, cecum of 0% HILM; CB, cecum of 1% HILM; CC, cecum of 3% HILM; CD, cecum of 5% HILM. X- and Y-axes represent two selected main coordinate axes, and percentage represents interpretation value of main coordinate axis to difference of sample composition; scales of the X- and Y-axes are relative distances and have no practical significance; points of different colors or shapes represent samples of different groups; the closer the two sample points are, the more similar the species composition of the two samples.

To analyze the effects of HILM on the microbial community, principal component analysis (PCA) used weighted UniFrac distances, which were calculated based on the OTU species and relative abundance of the samples (Figure 6). The results displayed significant differences between microbial communities in group C and group D of the jejunum but no significant differences between the microbial communities of groups A and B (Figure 6A). It suggested that the feed additives significantly influenced the gut microbiota distribution in chicken. Figure 6B showed that the degree of clustering of individuals in groups A and B was high, whereas the degree of clustering of individuals in groups C and D was low, indicating that the similarity within groups A and B was higher than in other groups. There were no significant differences among the microbial communities in the cecum (*p* > 0.05).

## Discussion

HILM contains higher or similar protein content and richer essential amino acid content (Yu et al., 2020). During recent decades, many studies indicated that the significant effects of HILM on intestinal health (Weththasinghe et al., 2021). Intestinal morphology and intestinal microbiota are critical to maintaining ecosystem stability and performance. HILM is a reasonable protein feed additive. The current study evaluated the effect of HILM on villus morphology of the jejunum and duodenum and the comparison of OTUs of microorganisms, alpha diversity, PCA cluster analysis, and microbial species composition analysis of the jejunum and cecum.

The rapid growth of broilers was due to the great potential of the small intestinal cells to absorb nutrients and the efficient conversion of nutrients to muscles (Mishra and Jha, 2019; Liu et al., 2020). Modifications of intestinal morphology, mainly increasing VH and the VH/CD in the duodenum and jejunum, could improve nutrient absorption (Schiavone et al., 2018; Hajimohammadi et al., 2020). Small intestinal VH determined the functional maturity of villus absorptive cells while the VH increases; the intestinal surface area enabled more efficient absorption of available nutrients (Li et al., 2019; Ramlucken et al., 2019). In this study, the supplementation of 1% HILM significantly increased the VH of the duodenum, and dietary 1 and 3% HILM supplementation had no damage effect in the duodenum and jejunum. In conclusion, dietary supplementation of HILM could improve nutrient absorption, maintain the normal intestinal morphology, and had effects on VH, CD, and VH/CD. However, dietary supplementation of 5% HILM reduced the VH of the jejunum and duodenum and VH/CD of the jejunum, consistent with the adverse effects of protein source substitution on small intestinal morphology in broilers (Biasato et al., 2018b). Interestingly, the CD of dietary 5% HILM supplementation in the jejunum decreased, which contrasted with earlier studies (Ibitoye et al., 2019). This might be related to the high chitin content in HILM, which may cause damage to the intestinal structure and reduce nutrient digestibility (Kroeckel et al., 2012). The supplementation of chitinase in diets supplemented with HILM might improve the effect, which needed to be studied in the future.

The intestinal tracts of poultry were complex, diverse, and dynamic microbial communities, which played an important role in host health and production performance. In the process of nutrition, metabolism, physiology, and immunity, the microbial community could promote digestion and absorption of nutrients, stimulate the immune response of the host, and enhance resistance to infection (Shi et al., 2020). However, gut microbiota also had direct and indirect harmful effects on decreased digestibility, increased cell turnover rate, and production of toxic metabolites, which might also lead to the decreased growth performance of chickens (Wang et al., 2017; Yadav and Jha, 2019). It was necessary to sufficiently understand the composition and diversity of the intestinal microbiome to further promote poultry growth and intestinal health. In this study, 16S recombinant DNA gene sequencing was used to detect the microbial diversity and community compositions of bacteria in the jejunum and cecum of Xuefeng black-bone chickens fed with different doses of HILM diets. The OTU number of dietary 3% HILM supplementation was the highest in the jejunum, which implied that the 3% HILM to the diet increased the microbial diversity of the jejunum. However, none of the groups in both the jejunum and cecum had the unique OTU number, indicating that dietary HILM had no effect but maintained species richness.

In different parts of the intestine, due to different intestinal structures, pH values, and diet status, bacterial populations vary greatly. Alpha diversity analysis analyzed species diversity in independent samples (Cui et al., 2021). The high diversity of intestinal bacteria is conducive to maintaining intestinal stability. In this study, we investigated the ACE, Chao, Shannon, and Simpson indexes in the jejunum and cecum of the dietary HILM to Xuefeng black-bone chickens. The results showed that dietary 3 and 5% HILM supplementation significantly increased the ACE and Chao indexes, and 1% HILM supplementation significantly decreased the Simpson index in the cecum, consistent with the report that highly nutritious diets could increase diversity indexes (He et al., 2020). Although there were no significantly differences of alpha diversity index in the jejunum, 3% HILM had the highest ACE, Chao, and Shannon indexes and the lowest Simpson index. Accordingly, the alpha diversity index was used as the community richness index. The higher the ACE, Chao, and Shannon indexes, the higher the community richness, whereas the Simpson index was the opposite. Improving the alpha index of dietary HILM might increase the stability between communities and closer the connections between communities.

Robust and balanced compositions of intestinal microflora were required to support health and growth. Microorganisms could benefit the host by affecting nutrient digestion and disease resistance, but an overgrowth of gut microbial could affect ecosystem balance and destroy gut integrity to initiate intestinal inflammation (Saracila et al., 2018). HILM, as a kind of feed resource based on insects, which could maintain intestinal microorganism health, has been reported in animal feed production (Leni et al., 2020). Based on the results of species annotation, we analyzed the microbial composition of the cecum and jejunum at the phylum and genus levels to evaluate the role of HILM in maintaining homeostasis.

At the phylum level of the jejunum and cecum microbial composition, both *Bacteroidetes* and *Firmicutes* were the largest phyla, which accounted for >80% of all the microbial community detected in our study, and *Bacteroidetes* and *Firmicutes* accounted for >90% in the cecum. This was consistent with many previous studies suggesting that *Firmicutes* and *Bacteroidetes* made up the majority of the microbial communities in chickens (accounting for >80%) at the phylum level, and these bacteria affected energy production and metabolism (Shang et al., 2018; Herrero-Encinas et al., 2020). *Bacteroidetes* and *Firmicutes* were the common colonizers of the chicken intestinal, which were considered to be potentially beneficial autochthonous bacteria (Borda-Molina et al., 2020). Cui et al. (2021) found that these two kinds of a dominant phylum of the gut community played an important role in improving intestinal diseases, inhibiting the proliferation of harmful intestinal bacteria, and producing short-chain fatty acids, which reduced luminal pH and regulated the microbial composition (stimulating the growth of beneficial bacteria, such as *Bifidobacterium*) (Dong et al., 2017; Ratajczak et al., 2019). At the phylum level of the jejunum, we found that HILM supplementation decreased the number of *Deferribacterota* and *Fusobacteriota* phyla. *Deferribacterota* has been found in healthy mice and humans. However, the relatively small proportion of intestinal microorganisms and their role and function have been rarely reported (Lewis et al., 2021). Also, Fusobacteria, anaerobic Gram-negative rods, were rare agents of severe human diseases that had repeatedly noted their link to colorectal cancer. It was indicated that HILM supplementation to the diet could alleviate damage and improve the intestinal microbiota disorders caused by the disease. At the phylum level of the cecum, the *Campilobacterota* phyla were greatly improved with 3% HILM supplementation, which was not a usual report with the phylum by poultry studies under natural or captivity conditions. Although some members of this phylum might cause diseases in wild and domestic animals, they were considered to be nonpathogenic and were frequently isolated from healthy birds (Zhao Y. et al., 2019; Chen et al., 2020; Maraci et al., 2021). It was showed that HILM could affect intestinal microbiota, either directly or indirectly, by modulating jejunal and cecal microbial compositions at the phylum level, especially a diet with 3% HILM supplementation.

At the genus level, the dominant bacteria in the gut were remodeled. Different from phyla, the dominant taxa in chicken cecal microbiota was controversial (Biasato et al., 2018a). Costa et al. (2017) found that the most predominant genera in the cecum of broilers were *Clostridium*, *Ruminococcus*, *Lactobacillus*, and *Bacteroides*. Callaway et al. (2009) found that *Prevotella* was the most abundant genus. In particular, the *Bacteroides* genus has been reported as the most predominant member of the cecal microbiota of Bermuda free-range broilers. In this study, the genus *Bacteroides* was the positive bacterium in the jejunum and cecum, which is in line with the previous finding that a higher number of *Bacteroides* enables larger efficiency of gut microbiota in extracting energy from the diet (Yue et al., 2019). Of interest, in our findings, the genus *Lactobacillus* was the second leading bacterium in the jejunum. Li et al. (2020) also found that *Lactobacillus* consisted in the gut of healthy pigs. *Lactobacillus* had a strong antibacterial activity, which enhanced the health of the chickens by inhibiting the growth of pathogens through competitive exclusion in the gastrointestinal tract (Ahmed et al., 2019). In addition, dietary 3% HILM increased the genus *Romboutsia*, a valuable intestinal biomarker in maintaining the health of the host (Mangifesta et al., 2018; Zhang et al., 2021). However, the 5% HILM supplementation increased the genus *Staphylococcus* and decreased the genus *Megamonas* in the jejunum. *Megamonas* was a genus of Firmicutes bacteria, which has been reported that it acted as a hydrogen sink in the gut of broilers by increasing the production of short-chain fatty acids (Chen et al., 2019). In general, most *Staphylococcus* were nonpathogenic bacteria, but a few might cause disease. The result showed that high-level protein might result in the decrease of beneficial microbes, leading to an imbalance of the jejunum microbiota. Diet was one of the most important factors that affected the gut microbiota (Makki et al., 2018). The components of dietary protein affected the composition of gut microbiota, and higher levels could lead to an increase in pathogenic microorganisms, thereby increasing the associated risk of metabolic diseases (Zhao J. et al., 2019; Sedgh-Gooya et al., 2021). Moreover, in the cecum, the genus *Lactobacillus* was the second leading bacterium. Dalle Zotte et al. (2021) fattened quails with full-fat or defatted silkworm pupa meal to observe the cecal microbiome and found that the genus *Rikenellaceae* was increased, which was consistent with our results. *Rikenellaceae* has been reported to promote starch decomposition in raw potato starch-fed mice (Bang et al., 2019). Meanwhile, we observed that the genus unclassified *Bacteroidales* in 1% HILM of the cecum was significantly increased. The results showed that dietary 1 or 3% HILM could increase the number of beneficial microflora, maintaining the jejunal and cecal microflora stability. However, 5% HILM supplementation might have no beneficial effect on the intestine.

The main intention of PCA cluster analysis was to investigate the similarity of community structure among varied samples, to observe the differences among samples by sorting the samples by decomposing the community data structure (Fang et al., 2016). In this study, the weighted UniFrac plot showed that the jejunal microbial community of groups C and D was highly isolated from groups A and B. Cui et al. (2021) reported that the grouping effect was obvious and could effectively stabilize the progenitor of intestinal flora. It indicated that a diet supplemented with 3 and 5% HILM could change the species and abundance of main intestinal microflora and stabilize the composition of intestinal microflora. In addition, diet HILM had little effect on the difference of community members and the abundance of community members of the cecum.

Our previous data demonstrated that dietary supplementation of 3% HILM in broiler breeders could significantly increase feed intake and egg weight. Additionally, HILM could strengthen the activity of total-superoxide dismutase and the level of antibody to avian influenza virus of plasma. The data of this study displayed that dietary supplementation of 1 or 3% HILM benefited the intestinal morphology and intestinal microbiota of Xuefeng black-bone chicken. Together, 3% HILM dietary supplementation might improve intestinal morphology and regulate microbiota, increasing feed intake, egg weight, and disease resistance. Hence, HILM would be a suitable substitute for plant protein in the diet of Xuefeng black-bone chicken.

## Conclusion

Dietary supplementation of HILM to Xuefeng black-bone chicken diets improved intestinal morphology, alpha diversity, and beneficial flora content (*Campilobacterota*, *Romboutsia*, unclassified *Bacteroidales*). Among the three additive quantities, the addition of 1% HILM increased the VH of the duodenum and decreased the Simpson index of the cecum, and the addition of 3% HILM stabilized the composition of the jejunum. However, the addition of 5% HILM decreased the VH and the ratio of VH/CD of the jejunum and increased pathogenetic genera (*Staphylococcus*). Dietary 1 or 3% HILM might benefit intestinal morphology and intestinal microbiota of Xuefeng black-bone chicken. HILM could be an alternative protein supplement to meet the growing demand in poultry production.

## Data Availability Statement

The datasets presented in this study can be found in online repositories. The names of the repository/repositories and accession number(s) can be found below: https://www.ncbi.nlm.nih.gov/bioproject/PRJNA747901/.

## Ethics Statement

The animal study was reviewed and approved by the Animal Care and Use Committee of Hunan Agricultural University (HUNAU2020005).

## Author Contributions

CH, JC, and XZ designed the experiment. JL and YY performed the experiment. KX and XW collected the samples. QY, BX, XQ, and SG analyzed the samples and data. SG, CH, and XZ conceptualized the manuscript, compiled all of the information, and prepared the manuscript. All authors read and approved the final manuscript.

## Conflict of Interest

BX was employed by Hunan Yunfeifeng Agricultural Co. Ltd. The remaining authors declare that the research was conducted in the absence of any commercial or financial relationships that could be construed as a potential conflict of interest. The reviewer RF declared a shared affiliation, with no collaboration, with the authors, to the handling editor at the time of the review.

## Publisher’s Note

All claims expressed in this article are solely those of the authors and do not necessarily represent those of their affiliated organizations, or those of the publisher, the editors and the reviewers. Any product that may be evaluated in this article, or claim that may be made by its manufacturer, is not guaranteed or endorsed by the publisher.

## Footnotes

1
www.majorbio.com
